# Rhodopsins build up the birefringent bodies of the dinoflagellate *Oxyrrhis marina*

**DOI:** 10.1007/s00709-021-01717-y

**Published:** 2021-11-04

**Authors:** Erhard Rhiel, Christian Hoischen, Martin Westermann

**Affiliations:** 1 Plankton Ecology, Institute for Chemistry and Biology of the Marine Environment, Carl Von Ossietzky University Oldenburg, Carl–von–Ossietzky–Straße 9–11, D–26129 Oldenburg, Germany; 2 Electron Microscopy Center at the Jena University Hospital, Friedrich–Schiller–University Jena, Ziegelmühlenweg 1, D–07743 Jena, Germany; 3 CF Imaging, Leibniz Institute On Aging, Fritz–Lipmann–Institute (FLI), Beutenbergstraße 11, D–07745 Jena, Germany

**Keywords:** Birefringent bodies, Dinoflagellate, Freeze-fracture electron microscopy, *Oxyrrhis marina*, Rhodopsins, Transmission electron microscopy

## Abstract

The ultrastructure of the birefringent bodies of the dinoflagellate *Oxyrrhis marina* was investigated by transmission electron microscopy. Ultrathin sectioning revealed that the bodies consist of highly ordered and densely packed lamellae, which show a regular striation along their longitudinal axis. A lattice distance of 6.1 nm was measured for the densely packed lamellae by FFT (Fast Fourier Transformation) analysis. In addition, a rather faint and oblique running striation was registered. Lamellae sectioned rather oblique or almost close to the surface show a honeycombed structure with a periodicity of 7.2–7.8 nm. Freeze-fracture transmission electron microscopy revealed that the lamellae are composed of highly ordered, crystalline arrays of particles. Here, FFT analysis resulted in lattice distances of 7.0–7.6 nm. Freeze-fracture transmission electron microscopy further revealed that the bodies remained intact after cell rupture followed by ascending flotation of the membrane fractions on discontinuous sucrose gradients. The birefringent bodies most likely are formed by evaginations of membranes, which separate the cytoplasm from the food vacuoles. Distinct, slightly reddish-colored areas, which resembled the birefringent bodies with respect to size and morphology, were registered by bright field light microscopy within *Oxyrrhis marina* cells. An absorbance maximum at 540 nm was registered for these areas, indicating that they are composed of rhodopsins. This was finally proven by immuno-transmission electron microscopy, as antisera directed against the C-terminal amino acid sequences of the rhodopsins AEA49880 and ADY17806 intensely immunolabeled the birefringent bodies of *Oxyrrhis marina*.

## Introduction

Fifty years have passed by since the first description of cellular structures which to date have solely been described for *Oxyrrhis marina*, the birefringent bodies (Dodge and Crawford 1971). In the light microscope, the bodies are highly birefringent and fusiforme. Transmission electron microscopy of ultrathin-sectioned cells revealed that the bodies show a variety of appearances, i.e., (1) crystalline bodies, showing a lattice being oriented parallel to the long axis of the structures; (2) less regularly structures that seem to shed the outer layers (fraying form); and (3) bodies, which dissociate into complex cisternae. The cisternae are enclosed by two layers, which the authors assumed to represent membranes. Most of the bodies measure 2 µm in size, but some up to 5 µm length were also registered. According to Dodge and Crawford (1971), the bodies are located in the cell vacuoles and are not surrounded by cytoplasm or a membrane. As the bodies somewhat resemble myelin sheaths, the authors supposed them to be composed of phospholipid material or contain phospho-lipoprotein, and that in this case, the bodies would function as a reserve for forthcoming membrane syntheses. The authors finally state that the bodies also may simply represent accumulated waste products.

Since then, research on the birefringent bodies of *Oxyrrhis marina* fell silent, although they are, admittedly rarely, documented in literature showing electron microscopic micrographs. Thus, they most likely can be seen in Fig. 2 of Kato et al. (2000) and in Fig. 16 of Jeong et al. (2008). Recently, Ammermann et al. (2017) investigated the predatory behavior of *Oxyrrhis marina* feeding on the prasinophyte *Pyramimonas grossii* and registered the birefringent bodies, too. The authors observed exclusively the fraying form. Most of the bodies were of elongated shape, but some resembled rather sickles or rings.

The current study continues on the observations of Dodge and Crawford (1971) and Ammermann et al. (2017) and tries to shed light on some of the still open questions. First, the ultrastructure of the birefringent bodies of *Oxyrrhis marina* was examined in more detail by regular transmission electron microscopy of ultrathin-sectioned and freeze-fractured cells. They were further investigated after cell rupture followed by ascending flotation of the membrane fractions on discontinuous sucrose gradients. Second, we scrutinized the origin of the birefringent bodies by ultrathin section transmission electron microscopy. Third, we recorded absorbance spectra of distinct areas within *Oxyrrhis marina* cells appearing reddish in bright field light microscopy and which we assumed to represent those bodies. Finally, fourth, we show by means of immuno-transmission electron microscopy that at least two rhodopsins constitute the birefringent bodies of *Oxyrrhis marina*.

## Material and methods

### Strain sources and growth conditions of stock cultures

*Oxyrrhis marina* was obtained from the Culture Collection of Algae at Göttingen University (strain B21.89; SAG, University of Göttingen, Germany). Stock cultures were grown in f/2 medium (Guillard and Ryther 1962) at 17 °C in Erlenmeyer flasks of 50–1000 ml culture volume without aeration. They were fed with dried yeast cells obtained from a local grocery store. Transfer into fresh medium occurred in intervals of 2 weeks. The photon flux density was adjusted to 3 μmol photons m^−2^ s^−1^ (measured with an Almemo 2290–2 measuring instrument equipped with a FLA613–PSM sensor, Ahlborn Mess– und Regelungstechnik GmbH, Holzkirchen, Germany). The light/dark regime was 14 h:10 h. For the experiments, the cultures of *Oxyrrhis marina* were fed either with dried yeast cells as described by Heyerhoff et al. (2019) or with *Pyramimonas grossii* as described by Ammermann et al. (2017) and used 7–8 days after adding the prey organisms.

### Cell disruption and membrane preparation procedures

Cells were disrupted according to Rhiel et al. (2020a), and membrane fractions were purified by ascending flotation on discontinuous sucrose gradients in the style of Chua and Bennoun (1975). Briefly, the cells (1000 ml culture volume) were harvested by centrifugation at 900xg for 15 min at 19 °C (Eppendorf 5810R; Eppendorf, Hamburg, Germany) using a swinging bucket rotor (A‐4‐62; Eppendorf). The resulting cell pellet was suspended in 1–4 ml f/2 medium and transferred into one to four 5 ml reaction tube(s) (Eppendorf). Trichocyst discharge was induced by the addition of 4 ml of distilled water containing 1.25% (w/v) ethylenediaminetetraacetic acid per 1 ml of concentrated *Oxyrrhis marina* cells. After incubating for 5–10 min, the cells were pelleted by centrifugation (200xg, 10 min, 19 °C), and the resulting supernatant was discarded. The centrifuged cells were dissolved in 1 ml 10 mM Tris/HCl buffer pH 7.5 containing 5 mM MgCl_2_ and either immediately processed further or kept frozen until use. Those cell suspensions were transferred into 2 ml rupture tubes (MP Biomedicals, Santa Ana) containing approximately 0.6 g of silica beads (0.1 mm in diameter, MP Biomedicals). Cell rupture was achieved using a FastPrep–24 5G bead beater (MP Biomedicals). Two treatments, each for 30 s and at 7 m s^‐1^, were conducted with a pause of 90 s on ice in between. Then, the glass beads were allowed to sediment, and the supernatants were transferred into fresh 5 ml reaction tubes. The glass beads were washed twice with 1 ml 10 mM Tris/HCl buffer pH 7.5 containing 5 mM MgCl_2_ each, allowed to sediment, and the supernatants were added to the first ones, thus resulting in total volumes of 3 ml of red-colored membrane fractions. The membrane fractions were centrifuged (3220 × g, 10 min, 10 °C), and the pelleted materials were dissolved in 1 ml of 10 mM Tris/HCl buffer pH 7.5 containing 1.8 M sucrose and 5 mM MgCl_2_. MgCl_2_ was added as it is known to stabilize stacked membranes. The membrane fractions were transferred into 13.5 ml ultra-clear centrifuge tubes (Beckman Coulter, Krefeld, Germany) and overlaid by 3 ml each of 1.5 M, 1 M, and 0.5 M sucrose dissolved in 10 mM Tris/HCl buffer pH 7.5, containing 5 mM MgCl_2_. The tubes were centrifuged (71015xg, 2 h, 8 °C) in an ultracentrifuge (L8‐55 M ultracentrifuge; Beckman) using a swinging bucket rotor (SW40; Beckman). The gradients were documented by photography and the bands were withdrawn using a syringe. Aliquots of the bands were used for freeze-fracture transmission electron microscopy (see below).

### Ultrathin sectioning transmission electron microscopy

The cells from 400–500 ml of culture medium were harvested by centrifugation (10 min, 900 × g; Eppendorf 5810R refrigerating centrifuge equipped with an A–4–62 swinging bucket rotor). The cells were then fixed for 2 h at room temperature using 4% (v/v) glutardialdehyde in f/2 medium containing 20 mM Na–K phosphate buffer (pH 7) and 0.05% (w/v) ruthenium red. Post-fixation was carried out for 1.5–2 h in 1% (v/v) OsO_4_. Then, the preparations were dehydrated in a graded ethanol series, with the 50% ethanol step containing 2% (w/v) uranyl acetate. Following dehydration, the samples were embedded in epoxy resin according to Spurr (1969). Ultrathin sections were cut with an Ultracut E microtome (Reichert, Germany). Samples were examined with a Zeiss EM 900 electron microscope (Zeiss, Oberkochen, Germany) operated at 80 kV. Digitized images were taken with a wide-angle dual speed 2 K CCD camera controlled by the Sharp/Eye base controller and operated by the Image SP software (TRS, Moorenweis, Germany). Lattice analyses were performed using the Fast Fourier Transformation (FFT) function of the ImageJ (v1.53c) software package (Abràmoff et al. 2004; https://imagej.nih.gov/ij/index.html).

### Freeze-fracture transmission electron microscopy

Cells from 40–50 ml of culture medium were concentrated by centrifugation (10 min, 5000 × g; Thermo Scientific Heraeus Biofuge stratos centrifuge, Fisher Scientific GmbH, Schwerte, Germany) at room temperature. Small aliquots of the pelleted cells (1–2 μl) were enclosed between two 0.1 mm thick copper profiles as used for the sandwich double-replica technique. Rapid plunge-freezing and freeze-fracturing followed the protocol outlined by Ammermann et al. (2013). The obtained replicas were transferred to a Proteinase K cleaning solution (400 μg/ml proteinase K, 0.5% (w/v) SDS, 0.5% (w/v) CaCl_2_, Tris/HCl 50 mM, pH 7.5) at 60 °C for 1 h, followed by a treatment in 65% (v/v) nitric acid for 15 min at 60 °C. Then, the replicas were washed three times in distilled water and transferred onto uncoated copper grids for examination in a Zeiss EM 900 electron microscope. Image acquisition and Fast Fourier Transformation (FFT) were performed as described above.

### Antisera production

The antisera directed against the C-termini of rhodopsins ADY17806 and AEA49880 of *Oxyrrhis marina* were done by a commercial facility (BioScience, Göttingen, Germany). For this, synthetic polypeptides were synthesized. The amino acid sequences of the oligopeptides were NAKSRLEEEGKLRA for ADY17806 (according to Slamovits et al. 2011) and NDDLLHVAMPTGVVEQ for AEA49880 (Slamovits and Keeling 2010, https://www.ebi.ac.uk/ena/browser/api/embl/AEA49880.1?lineLimit=1000). Two mg of the peptides, linked to keyhole limpet haemocyanin (KLH), were used for the immunization of rabbits. Pre-immune sera were taken before the first immunizations. The pre-immune sera and antisera (final bleedings) were used for immuno-transmission electron microscopy. The specificities of the two antisera were tested by Western immunoblotting experiments, which are described in a separate manuscript (Westermann et al. submitted).

### Immuno-transmission electron microscopy

The cells from 800–1000 ml of culture medium were harvested by centrifugation (10 min, 900 × g; Eppendorf 5810R refrigerating centrifuge equipped with an A–4–62 swinging bucket rotor) and resuspended in 9.25 ml f/2 medium. Then, 9.25 ml fixative (4% (v/v) of formaldehyde in f/2 medium) was added. Fixation was carried out for 1 h and at room temperature. Then, the cells were washed in Tris-buffered saline (TBS: 10 mM Tris, pH 7.5, 150 mM NaCl). Post-fixation was carried out for 2 h in 0.25% (v/v) glutardialdehyde in TBS. The samples were washed three times in TBS plus 100-mM glycine for 10 min, 15 min, and 30 min. After the washing steps, the preparations were cooled to 4 °C and dehydrated in a graded ethanol series, with the 50% ethanol step containing 2% (w/v) uranyl acetate. Then, the samples were embedded in either Lowicryl K4M or LR White resin according to the manufacturer`s specifications. Ultrathin sections were cut with an Ultracut E microtome (Reichert, Germany) and placed onto copper grids. For immuno-transmission electron microscopy, the grids were floated for 30 min onto 50 µl drops of LBB (Labeling Blocking Buffer: 0.5% BSA, 0.5% gelatine, 0.005% Tween–20 in TBS) followed by an incubation of 1 h on 50 µl drops of either antisera or pre-immune sera (negative control). The sera were diluted 1:300 (ADY17806) and 1:100 (AEA49880) in LBB. Then, the grids were washed in 5 to 10 drops of LBB and incubated for 30 min in gold-labeled goat-anti rabbit IgG conjugates (British Biocell International, Cardiff, UK). The conjugates were diluted 1:50 in LBB, and the gold particles were 10 nm in diameter. After washing with 5–10 drops of TBS, the samples were examined with a Zeiss EM 900 electron microscope. Digitized images were taken as described above.

### Preparation of Oxyrrhis marina cells for light microscopy

For light microscopy, *Oxyrrhis* cells from 100–150 ml culture volume were harvested by centrifugation at 900 × g for 10 min in an Eppendorf 5810R refrigerating centrifuge equipped with an A–4–62 swinging bucket rotor (Eppendorf) and resuspended in 1 ml f/2 medium. Fixation was achieved by adding an equal volume of 8% (v/v) formaldehyde dissolved in f/2 medium to a final concentration of 4%. After an incubation time of 1 h at room temperature, the cells were washed in 20 mM Tris–HCl pH 7.5, 150 mM NaCl (TBS) twice for 5 min each. Small aliquots of cell suspensions were dropped on polylysin-coated microscope slides (Polysine Slides, Gerhard Menzel GmbH, Braunschweig, Germany). After cells were allowed to settle down for 10 min, the supernatant was carefully removed, and coverslips (thickness No. 1.5) were mounted using Prolong™ antifade reagent without DAPI (Thermo Fisher Scientific, USA).

### Brightfield microscopy

Brightfield microscopy was performed on an Axio Imager Z1 microscope (Zeiss) equipped with a TL LED lamp (Zeiss), an AxioCam MRc 5 color camera (Zeiss), and a Plan-Apochromat 100x/1.4 Oil DIC M27 objective (Zeiss). Images were acquired in ZEN 2.6 software (Zeiss). The light source intensity was set to 9.76 V and the exposure time was varied.

### Absorbance spectroscopy in birefringent bodies with laser scanning microscopy

Images for absorption spectra were acquired on a Leica TCS SP8 STED–3X equipped with an inverted microscope (DMI 8; Leica Microsystems, Wetzlar, Germany) and a 100 × STED objective (HC PL APO CS2 100 × 1.4 oil STED, Leica) using laser lines (20% intensity) of a pulsed laser (KIT WLL2 470–670 nm; output 70%) in the full range from 470–670 nm. For this, every 2 nm a scan was performed starting at 470 nm and finishing at 670 nm. Transmitted light was detected with a PMT in the transmission channel (gain = 367.5). This setup allowed to detect the intensity of the transmitted light at each wavelength between 470 and 670 nm and in each region of the scanned image and therefore also specifically in the birefringent bodies. Imaging speed was at 400 Hz and the pinhole set to 1 AU. The zoom varied according to the size of the analyzed cells between 3.4 and 4.9, and the pixel sizes were set to values between 70 and 80 nm. The intensities in the birefringent bodies (I(roi 1)) as well as in regions with comparable size inside (I(roi 2); an intracellular control region without birefringent bodies) and outside the cells (I_0_(roi 3); and a measure for the output intensity of the light) were analyzed in all obtained images taken at different wavelengths using Leica’s LASAF quantification tool. Absorbance of the birefringent bodies was calculated for each image obtained at the various wavelengths as follows: absorbance = log (I_0_(roi 3)/(I(roi 1)). Absorbance spectra were plotted using SigmaPlot 12.5. For calculation of the mean absorbance spectrum of 9 different birefringent bodies the individual spectra were normalized to 1.

The validity of the method was checked by measuring the absorbance of an extract containing phycoerythrin PE–565. The water-soluble extract was isolated by freeze-thawing concentrated cells of a limnic cryptophyte species, followed by pelletizing the membrane fraction by centrifugation and keeping the supernatant for the measurements. For absorbance measurements, 300 µl of the supernatant were transferred into a square field of a µ–Slide 8 well (thickness No. 1.5; ibidi, Martinsried, Germany). Using a 20 × objective (HC PL APO CS2 20 × 0.75 IMM, Leica) transmitted light was detected in the center of the well with a PMT in the transmission channel (gain = 358.5). Imaging speed was at 400 Hz and pinhole set to 1 AU. At zoom 6 and a format of 720 × 720 the resulting pixel size was 135 nm. Intensities in the phycoerythrin solution (I) as well as in distilled water (I_0_) as a measure for the output intensity of the light were analyzed in all obtained images taken at different wavelengths using Leica’s LASAF quantification tool. Absorbance was calculated for each scan as follows: absorbance = log(I_0_/I).

### Quantification of the immuno-label

The amounts of gold particles positioned on or tightly to distinct cell organelles were enumerated on digitized images. Then, the amounts of gold particles per µm^2^ were extrapolated.

## Results

### Conventional transmission electron microscopy

**Fig. 1:**
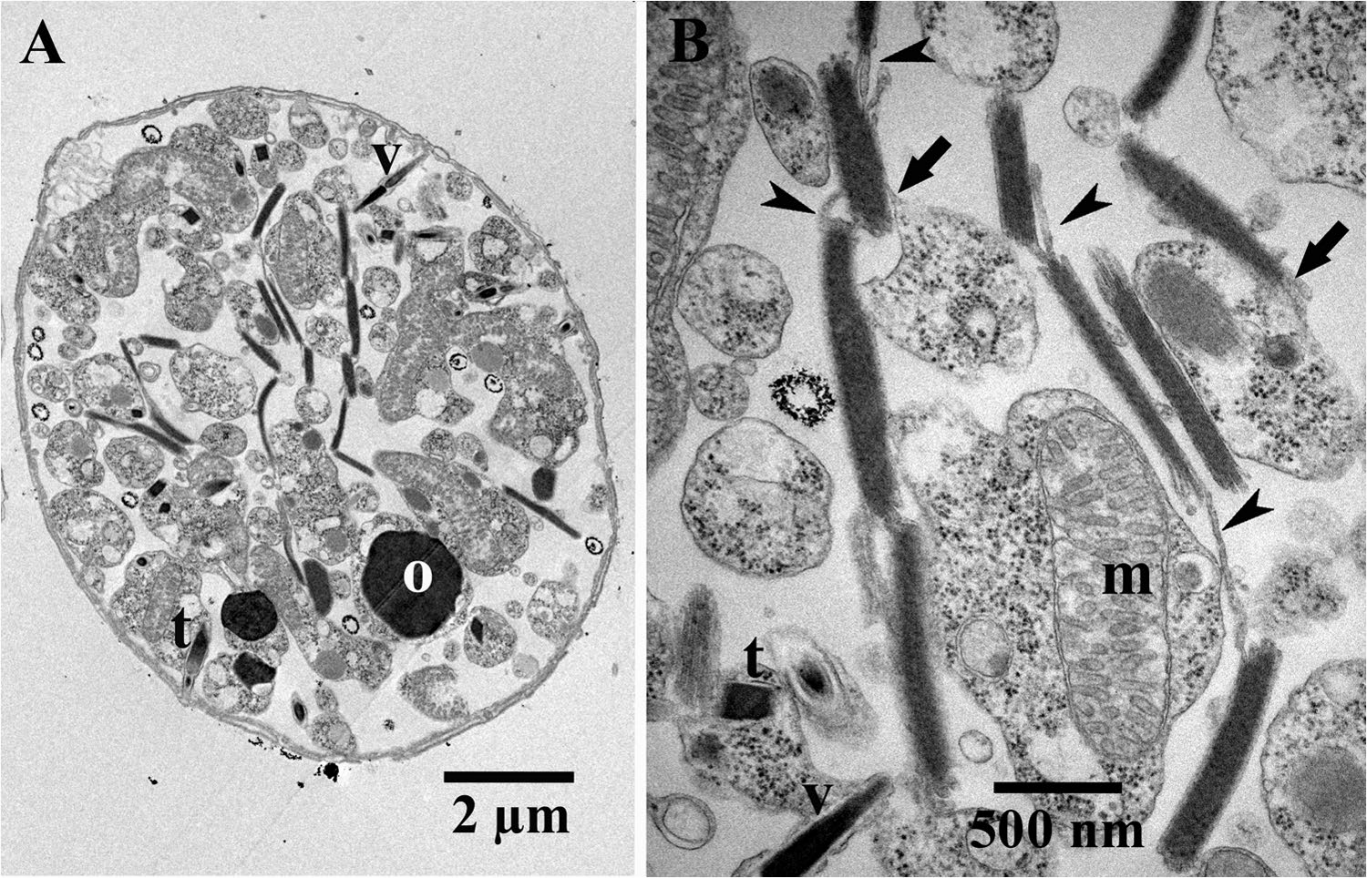
(**a**) Electron micrograph showing an ultrathin-sectioned cell of *Oxyrrhis marina*. (**b**) Micrograph showing the birefringent bodies in more detail. Some are connected among each other via membranes (indicated by arrowheads). Arrows indicate birefringent bodies, which are in close contact to the membranes encircling the cytoplasm. Abbreviations: m, mitochondrium; o, oil droplet; t, trichocyst; v, vlimatocyst. Scale bars are indicated

**Fig. 2:**
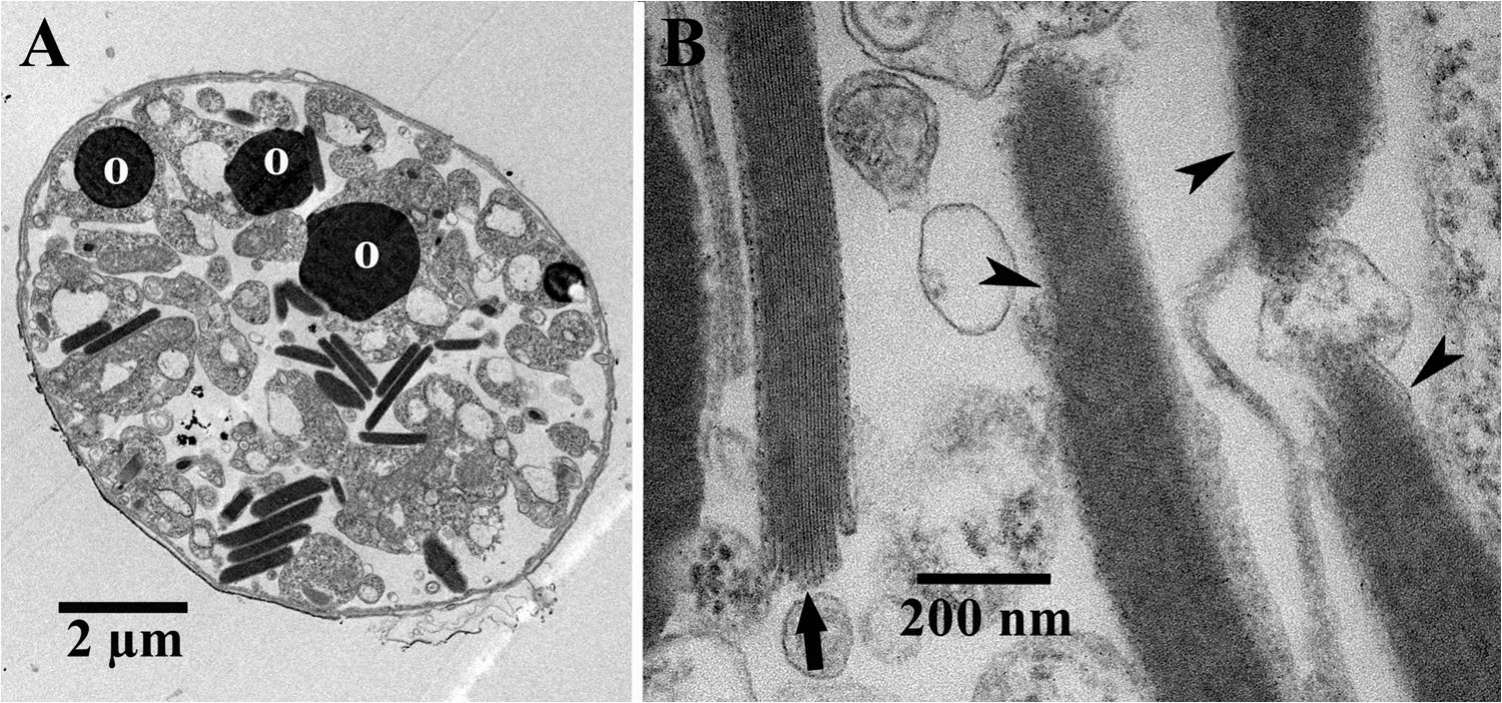
(**a**) Electron micrograph showing an ultrathin-sectioned cell of *Oxyrrhis marina*. (**b**) Micrograph showing the birefringent bodies in more detail. The directions of the rather faint and oblique running striations of three bodies are indicated by arrowheads; the longitudinal striation of a fourth body is marked by an arrow. Abbreviation: o, oil droplet. Scale bars are indicated

**Fig. 3:**
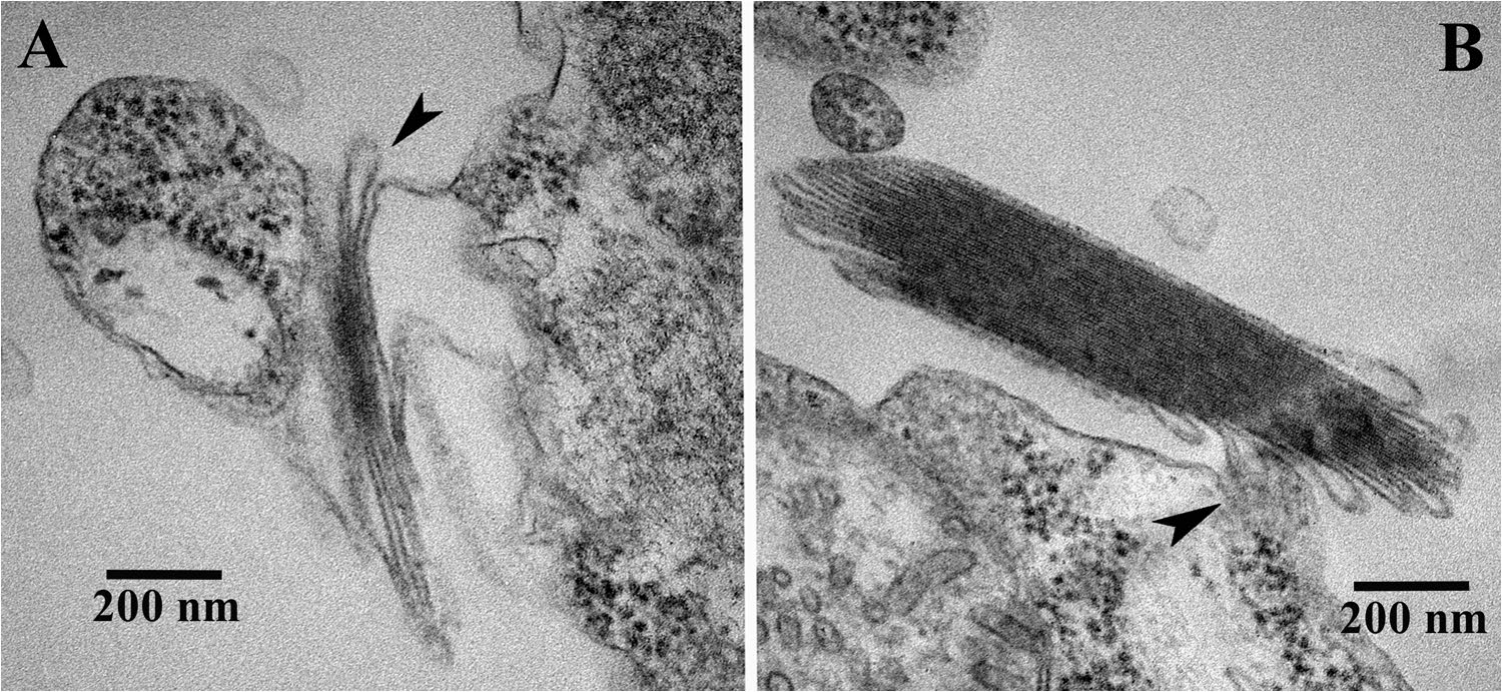
Electron micrographs showing most likely different steps of the development of the birefringent bodies and the connections of birefringent body membranes to neighboring membranes encircling the cytoplasm. In (**a**), only a few evaginations of the membrane encircling the cytoplasm can be seen (arrowhead), whereas in (**b**) many evagination loops have occurred. Scale bars are indicated

**Fig. 4:**
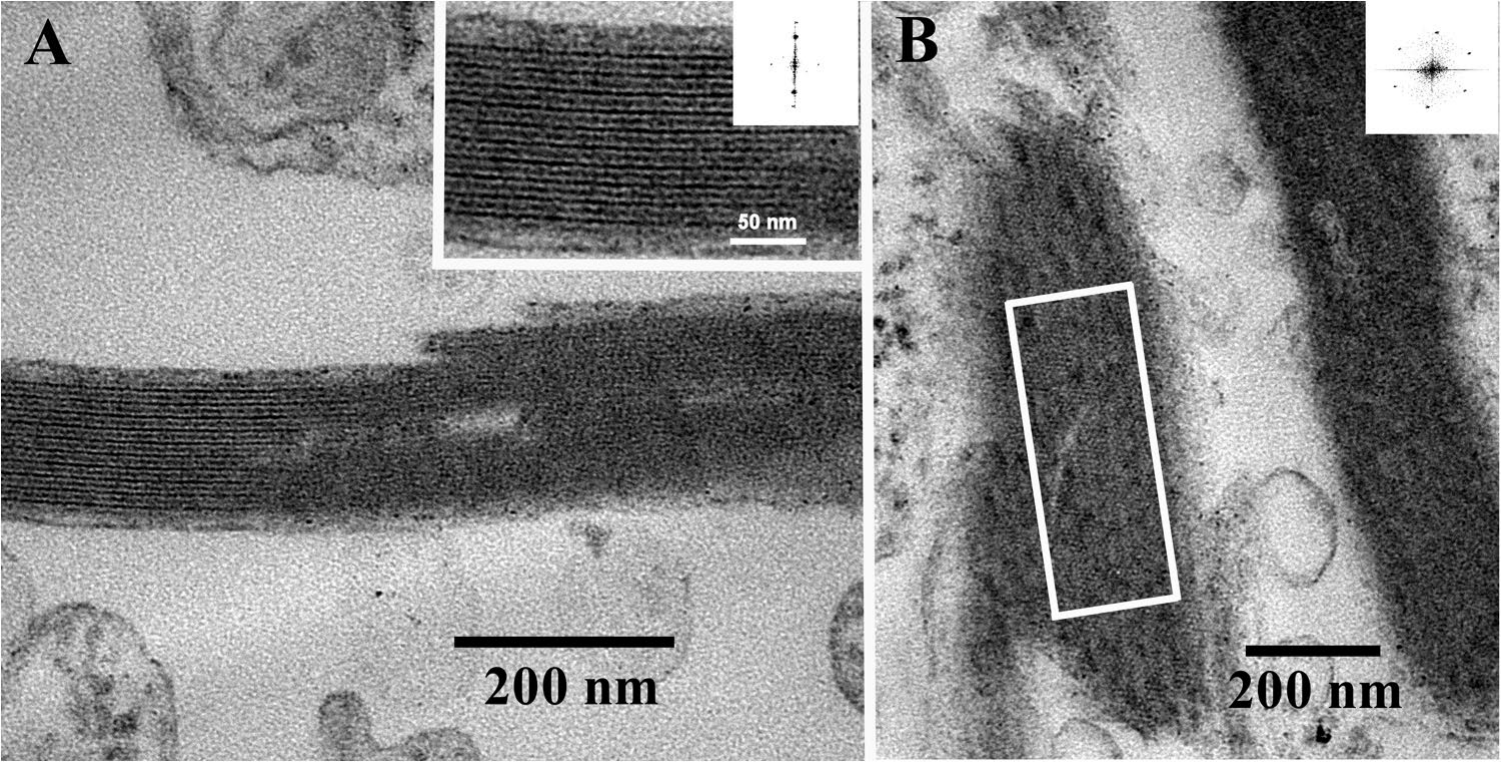
Electron micrographs of ultrathin–sectioned birefringent bodies. The one shown in (**a**) exhibits the longitudinal striation of the densely packed lamellae, whereas the one shown in (**b**) is sectioned rather oblique or almost close to the surface of the outermost lamella, showing a honeycombed structure. Inserts in (**a**) and (**b**) show the results obtained by FFT analyses of the areas marked by the squares. The FFT spectrum shown in (**a**) indicates a lattice distance of 6.1 nm, and the one shown in (**b**) indicates periodicities of 7.2, 7.5, and 7.8 nm. Scale bars are indicated

Ultrathin section transmission electron microscopy showed that birefringent bodies occurred in both, *Oxyrrhis marina* cells preying either on yeast or on *Pyramimonas grossii*. Exclusively the fraying form was observed (Figs. 1 and 2). The bodies resembled those already described by Ammermann et al. (2017), but seemed to occur more often and to be larger in length in cells preying on yeast. Up to 18 bodies were counted per sectioned *Oxyrrhis marina* cell fed on yeast, whereas only 2–3 were counted in sections of cells, which preyed on the prasinophyte. We passed on further quantification as it is impossible to deduce the total amounts of birefringent bodies per cell just from single ultrathin sections of cells, which had been cut in various planes and directions. The bodies were often in close vicinity to either lipid bodies or cytoplasm (Fig. 2) and connected among themselves via membranes (Fig. 1b). Both striations, the longitudinal striation of the membrane stacks and the rather faint and oblique running striation, were detected (Fig. 2b). Based on ultrathin sections of cells showing rather large amounts of birefringent bodies of various sizes and probably stages of development, it can be assumed that they derived by evaginations of membranes which separate the cytoplasm from the food vacuoles, so that they finally seemed to be located within the vacuoles (Fig. 3). They thus start with small amounts of meandering membranes (Fig. 3a) and grow and enlarge by the addition of further membranes (Fig. 3b). When cross-sectioned, the bodies appeared as highly ordered and densely packed lamellae, which show a regular striation of rather strong and weak electron-dense lines separated by electron-translucent lines along their longitudinal axis (Fig. 4a). FFT analysis and profile plots revealed a lattice distance of 6.1 nm (insert in Fig. 4a). In one instance, the lamellae of a birefringent body were sectioned rather oblique or almost close to the surface of the outermost lamella. They showed a honeycombed structure, indicating highly ordered arrays of the compounds constituting them (Fig. 4b). FFT analysis and profile plots revealed periodicities of 7.2, 7.5, and 7.8 nm for this lattice pattern (insert in Fig. 4b).

### Freeze-fracture transmission electron microscopy of cells

**Fig. 5:**
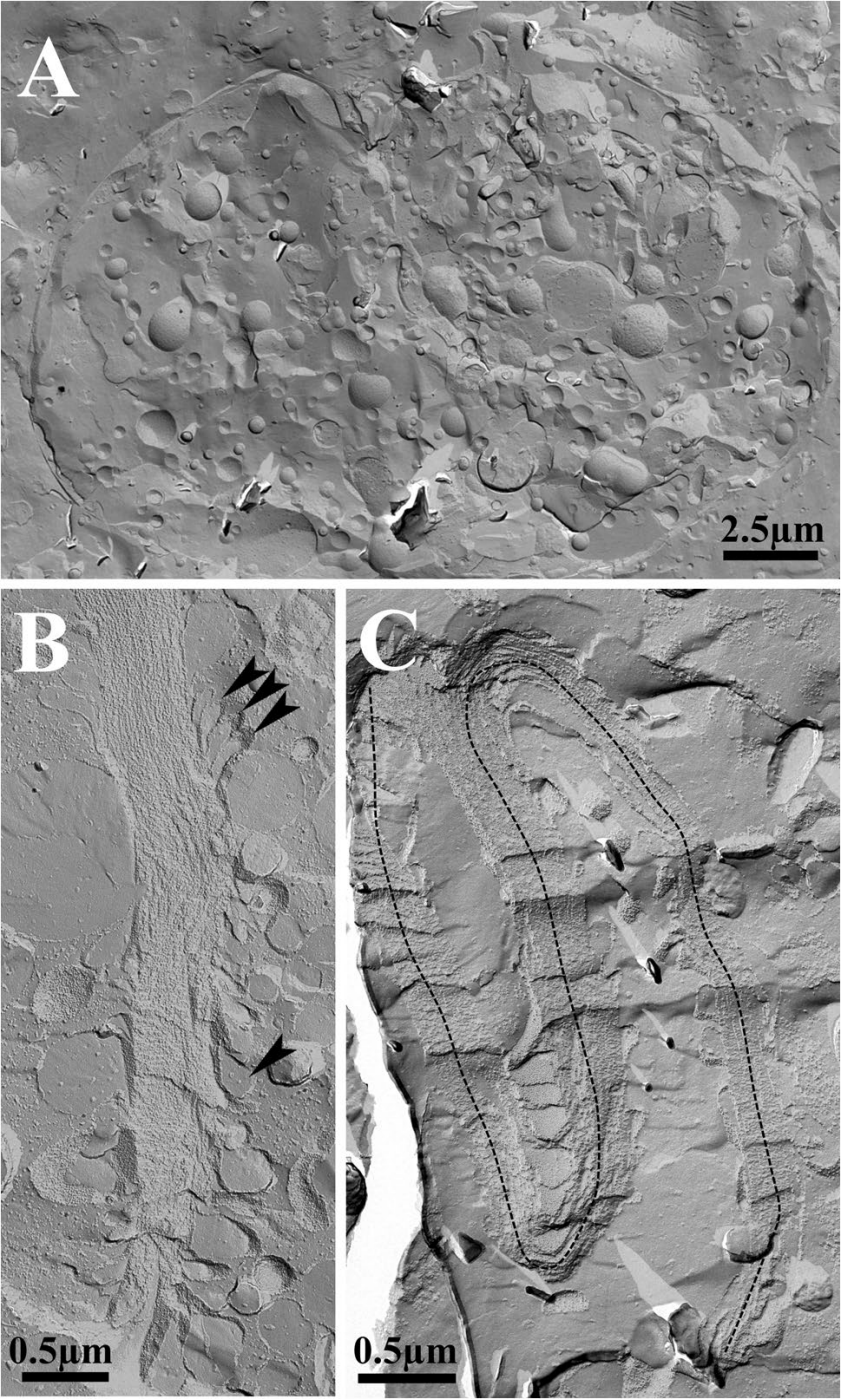
Electron micrographs showing a freeze-fractured *Oxyrrhis marina* cell (**a**) and birefringent bodies (**b**, **c**). The birefringent body shown in (B) consists of a single stack of densely packed lamellae. The arrowheads indicate membrane loops peeling off the birefringent body. The body shown in (**c**) seems to be bended twice, indicated by the dashed line, thus being composed of three bodies. Scale bars are indicated

**Fig. 6:**
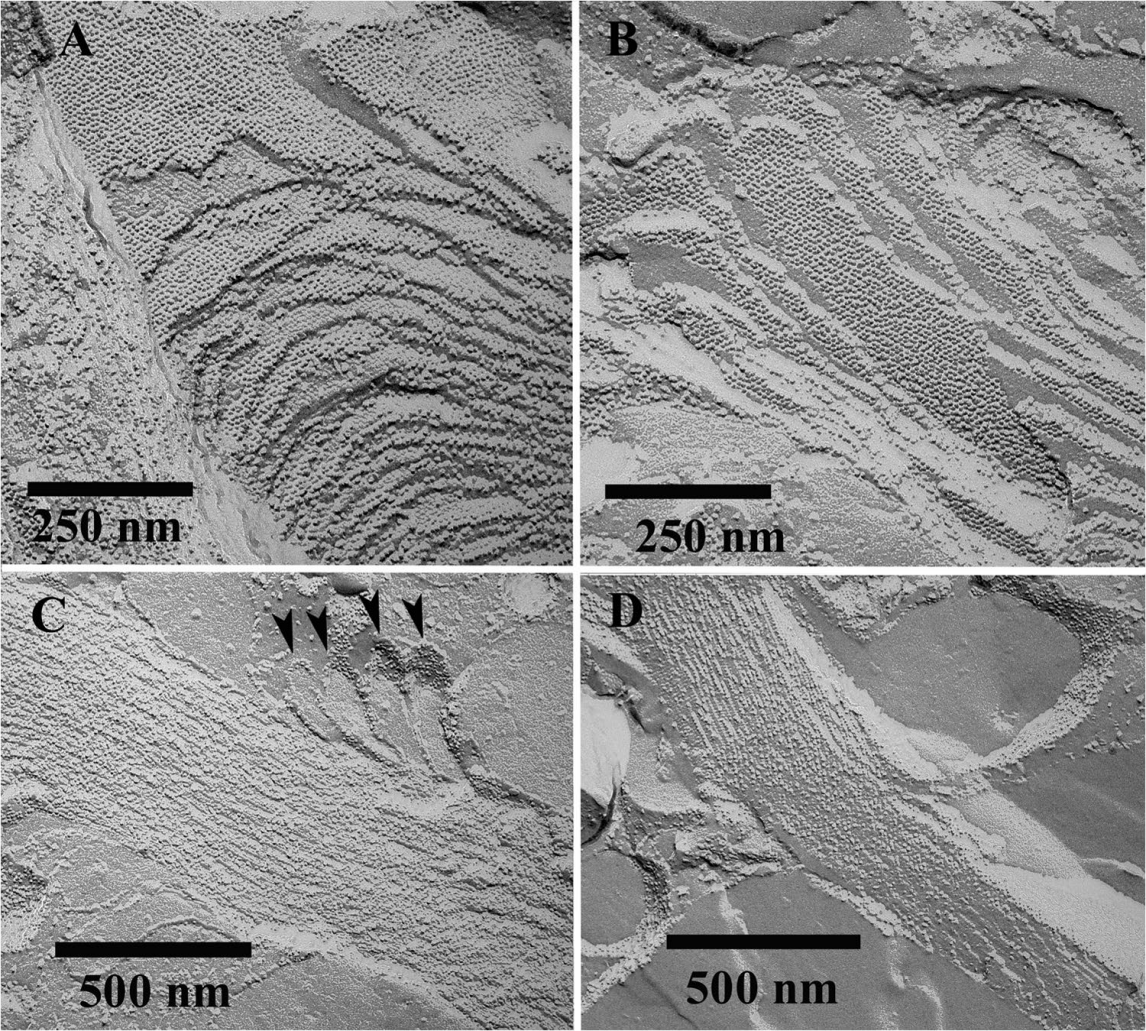
Electron micrographs showing freeze-fractured birefringent bodies. In (**a**) and (**b**) the bodies were fractured within the lamellae, whereas in (**c**) and (**d**) the densely packed lamellae have been cross-fractured. Note in (**c**) the loop structures, which seemed to peel off (marked by arrowheads). Scale bars are indicated

**Fig. 7:**
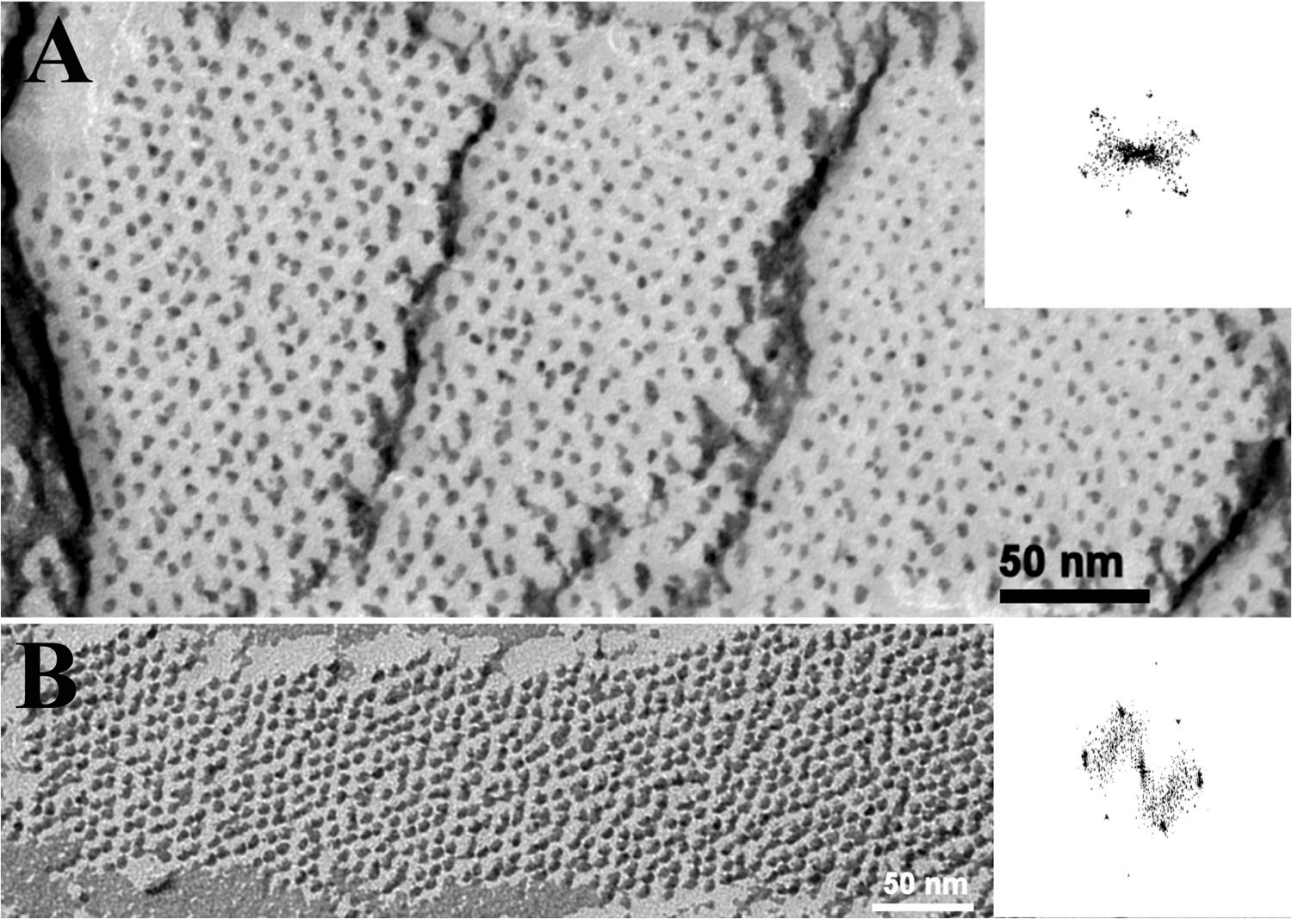
Electron micrographs showing freeze-fractured birefringent bodies, which had been subjected to FFT analyses. Lattice distances of 7.0–7.6 nm were calculated for these areas. Scale bars are indicated

In most of the cells, birefringent bodies were not seen by freeze-fracturing (Fig. 5a), and if so, they were hard to be detected at all (Figs. 5b, c). Cross-fractured bodies showed highly ordered and densely packed lamellae (Fig. 6c, d), with the edges of the bodies resembling membrane-like structures which form loops which seemed to peel off (Figs. 5b and 6c). Freeze-fracturing within the lamellae gave rise to highly ordered, crystalline arrays of particles (Fig. 6a, b). Here, FFT analysis revealed lattice distances of 7.0, 7.2, and 7.4 nm (Fig. 7b). Freeze-fracturing at the surface planes of the lamellae showed the arrays too, but less pronounced. FFT analysis revealed lattice distances of 7.4, 7.5, and 7.6 nm for these areas (Fig. 7a).

### Freeze-fracture transmission electron microscopy of isolated birefringent bodies

**Fig. 8:**
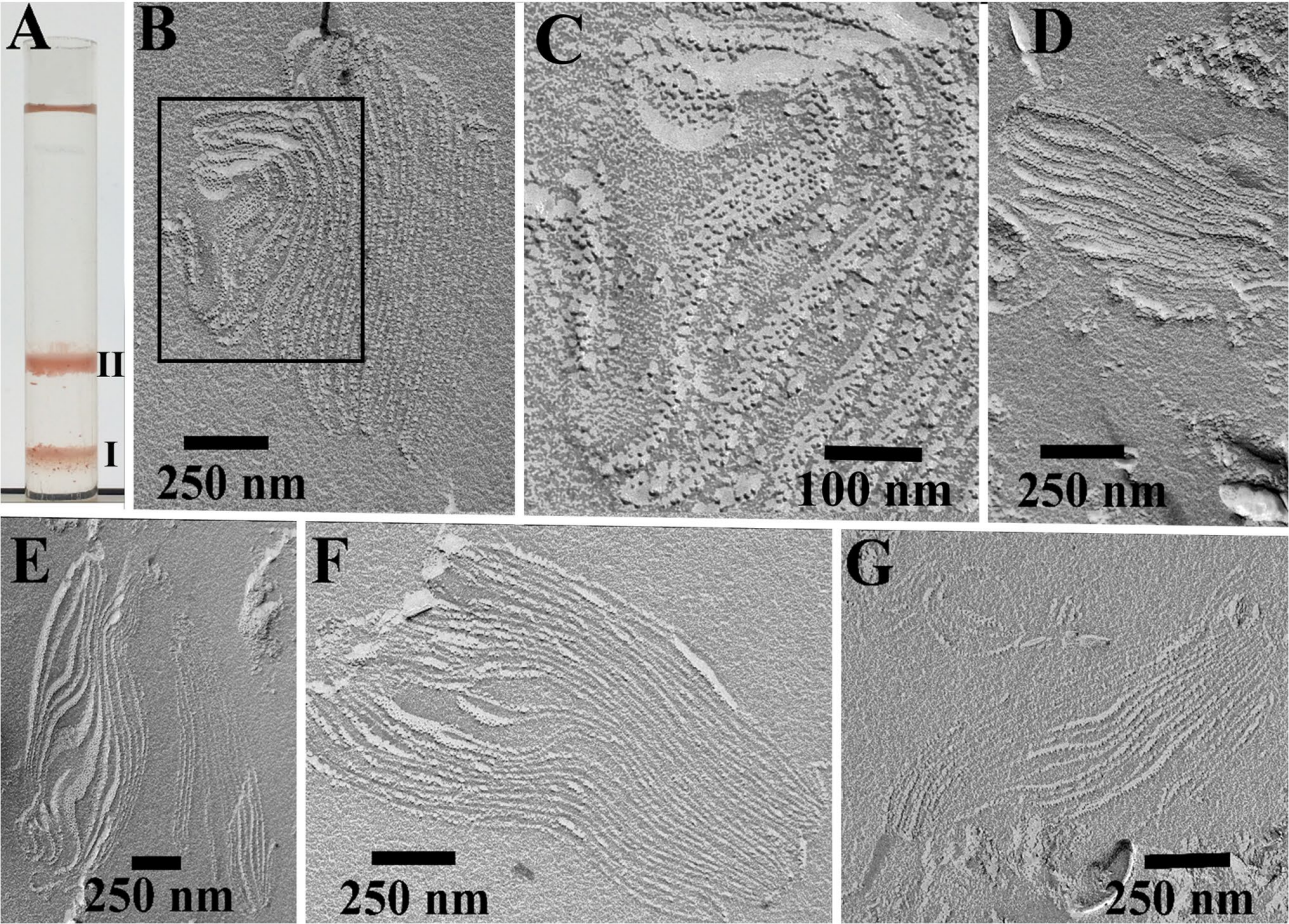
Electron micrographs showing freeze-fractured birefringent bodies, which had been isolated by ascending flotation on discontinuous sucrose gradients. A sucrose gradient is shown in A. The bands at the 1.8–1.5 M and 1.5–1.0 M sucrose interphases are labeled I and II. The pellet is not visible. Birefringent bodies, which have been fractured within the sheets are shown in (**b**),(**c**) (higher magnification of micrograph B), and (**e**) (on the left); rather cross-fractured birefringent bodies are shown in (**d**), (**e**) (on the right), (**f**), and (**g**). Scale bars are indicated

Either one band and a pellet or two bands and a pellet were obtained when membrane fractions of ruptured cells were subjected to ascending flotation on discontinuous sucrose gradients (Fig. 8a). The bands appeared at the 1.8–1.5 M (band marked I in Fig. 8a) and, in case of two bands, additionally at the 1.5–1.0 M sucrose interphase (band marked II in Fig. 8a). Subsequent freeze-fracture transmission electron microscopy revealed that the birefringent bodies within those bands were still intact, i.e., the lamellae remained densely stacked (Fig. 8d–g) and showed the crystalline arrays of particles (Fig. 8b, c).

### Absorbance spectroscopy using laser scanning microscopy

**Fig. 9:**
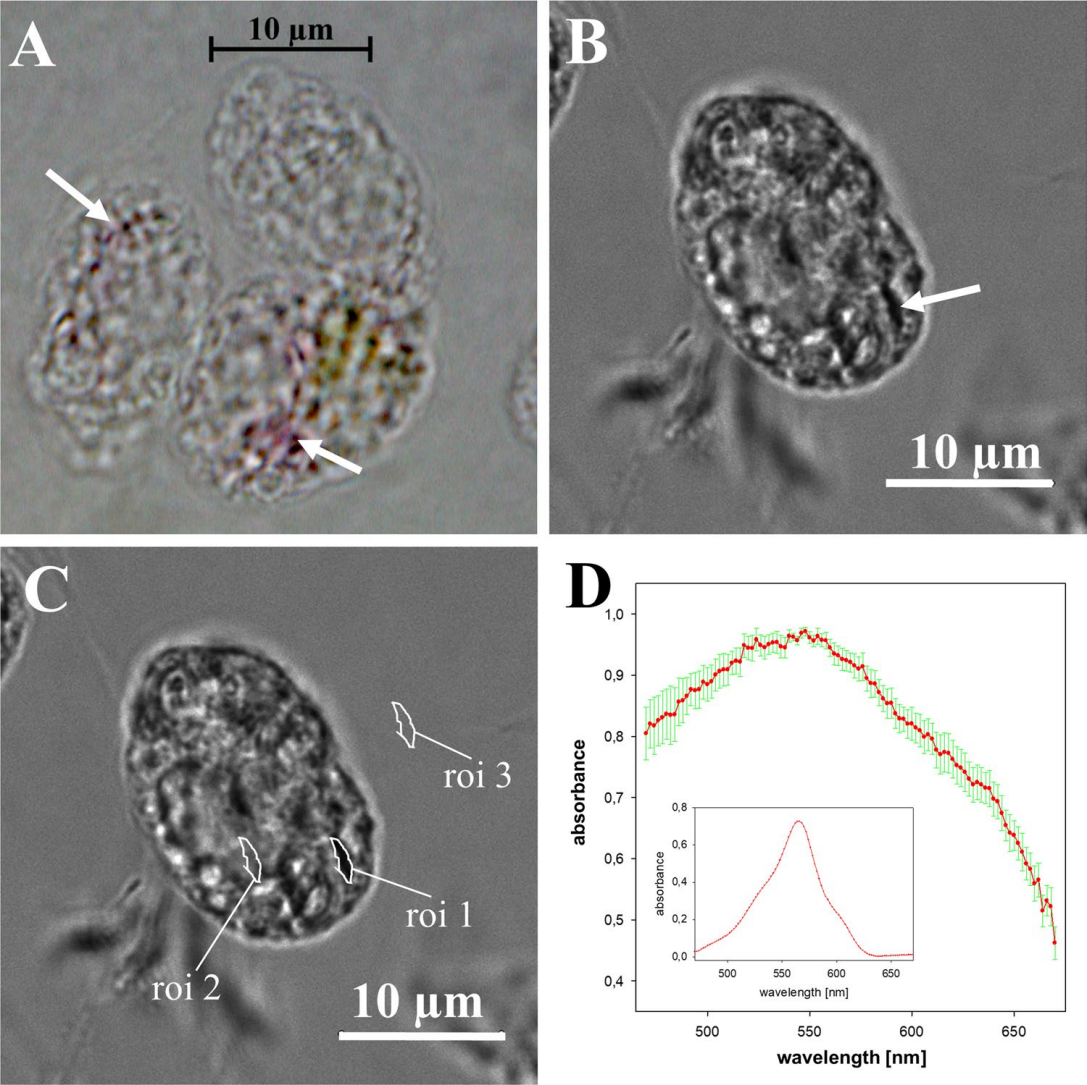
Light microscopical analysis of *Oxyrrhis marina*. (**a**) brightfield micrograph of *Oxyrrhis marina* cells illuminated with a Halogen lamp. The picture was taken with a color camera showing the natural staining of certain regions in the cell. Red areas, which presumably represent birefringent bodies are marked by arrows. In (**b**) is shown a laser scanning micrograph of an *Oxyrrhis marina* cell scanned at 550 nm with a transmitted light detector. This setup allows to detect the intensities of the transmitted light at each wavelength between 470 and 670 nm in each region of the cell and outside the cell. The arrow indicates the dark region with the size and the shape of a birefringent body. In (**c**) are indicated the regions where the intensities of the transmitted light were analyzed in scans performed every 2 nm. Roi 1 covers the area of the birefringent body, roi 2 covers a control region without birefringent body and the intensities determined in roi 3 served as a measure for the output intensities of the light. (**d**) is the mean absorbance spectrum of 9 birefringent bodies out of 8 cells after normalization to the value 1. The standard errors of the mean (SEM) are in green. The absorbance in the birefringent bodies was calculated with the formula absorbance = log (I_0_(roi 3)/(I(roi 1)). The inset shows the absorbance spectrum of a phycoerythrin-containing solution obtained with laser scanning light microscopy. Scale bars are indicated in (**a**), (**b**) and (**c**)

Occasionally small, slightly reddish areas were registered by bright field light microscopy within the cells (Fig. 9a). They measured up to 1 µm in length and appeared somewhat diffuse, birefringent, and tapering towards the ends (Figs. 9b and c). In some instances, one got the impression that they were connected among each other. The results of the absorbance measurements of those areas are compiled in Fig. 9d, with the spectrum representing the mean of nine measurements of nine individual reddish areas from eight different cells (exemplarily marked in roi 1 of Fig. 9c). A rather broad absorbance maximum at approximately 540 nm was determined. Absorbance spectra measured in intracellular regions without birefringent bodies (Fig. 9c, roi 2) never showed similarity to the spectra measured in the birefringent bodies (data not shown). Measurements of a water-soluble protein extract containing phycoerythrin PE–565 resulted in the expected absorbance spectrum with an absorbance maximum at 565 nm (inset in Fig. 9d).

### Immuno-transmission electron microscopy

**Fig. 10:**
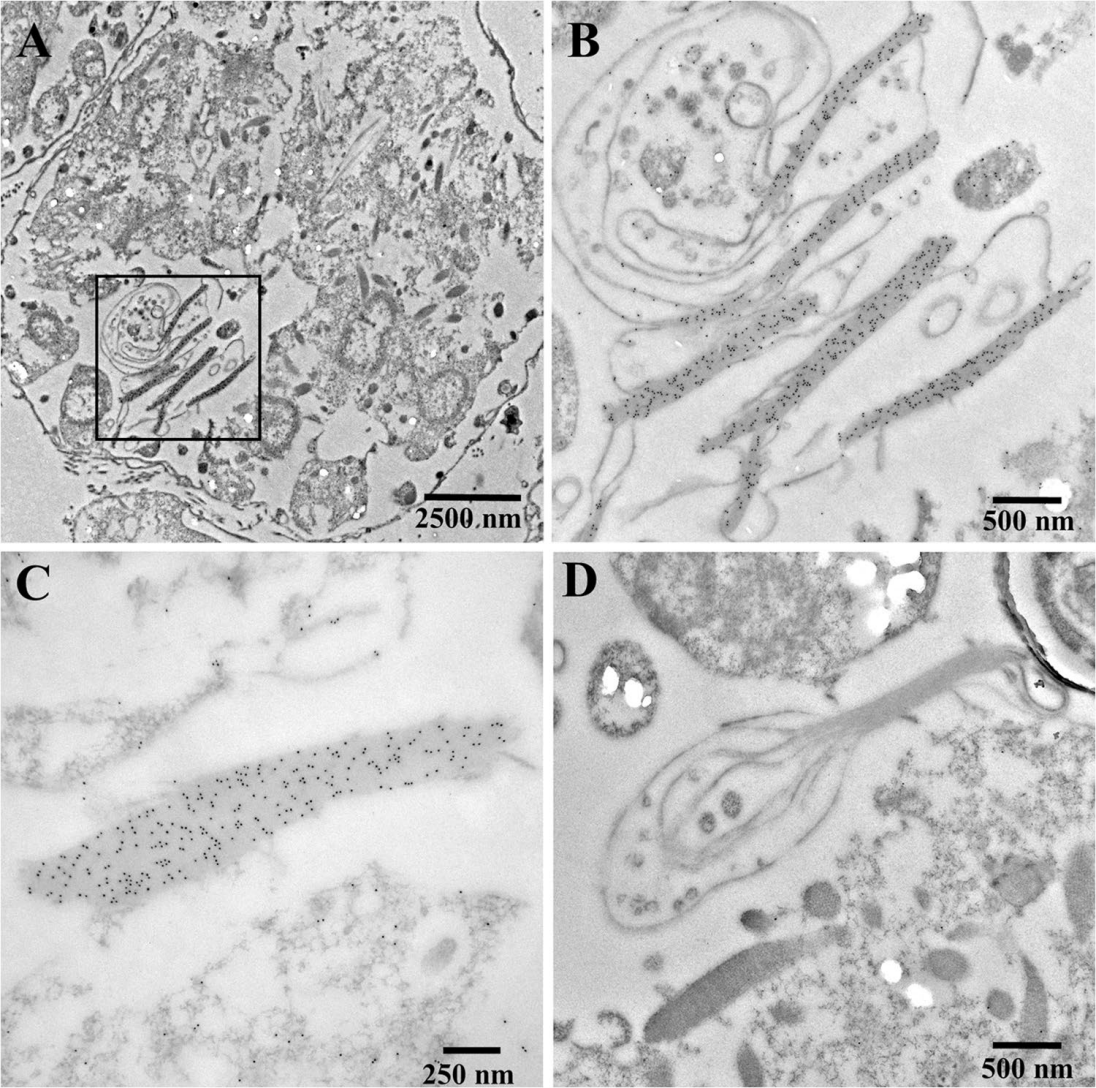
Electron micrographs of ultrathin-sectioned cells of *Oxyrrhis marina* embedded in LR White resin, labeled with the AEA49880 antiserum, showing an entire cell (**a**), a larger cell area (**c**), and a detail (**b**) of the area within the square marked in (**a**). All three micrographs show intensely labeled birefringent bodies. The pre-immune serum for AEA49880 (**d**) showed neither labeling on birefringent bodies nor on other cellular structures. The scale bars are indicated

**Fig. 11:**
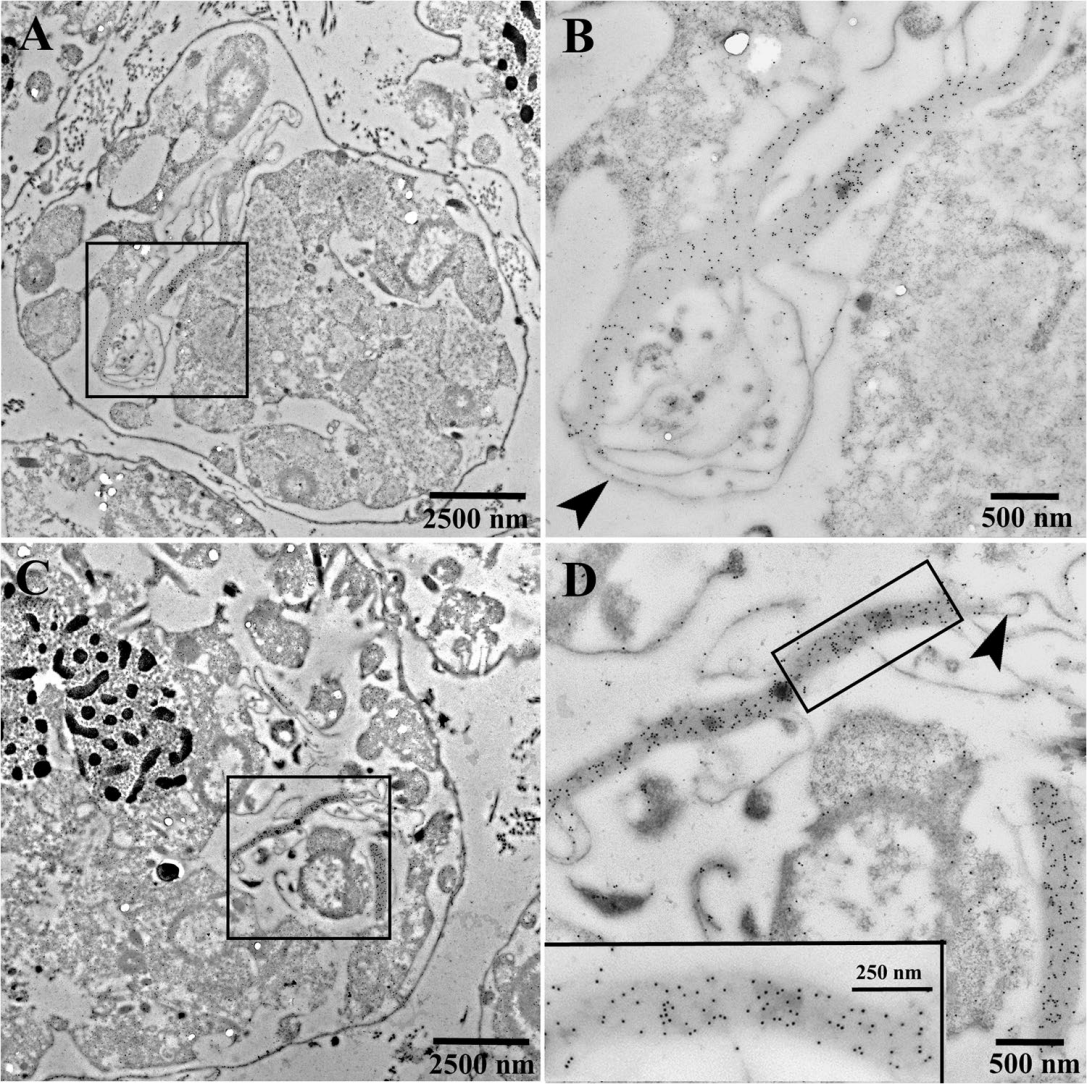
Electron micrographs of ultrathin-sectioned cells of *Oxyrrhis marina* embedded in LR White resin and labeled with the ADY17806 antiserum. An entire cell is shown in (**a**), a larger cell area in (**c**), and details in (**b**) and (**d**) of the areas within the squares marked in A and C. The bottom inset in (**d**) represents a detail view of the intensely labeled area of a birefringent body shown in the upper part of the micrograph. Arrowheads in (**b**, **d**) indicate membrane loops originating at the endings of the labeled structures. The scale bars are indicated

Micrographs obtained by immuno-transmission electron microscopy of cells of *Oxyrrhis marina* are shown in Figs. 10 and 11. The antiserum against AEA49880 (Fig. 10) and ADY17806 (Fig. 11) resulted in an intense labeling of the birefringent bodies. For the AEA49880 antiserum, mean values of 10 and 338 gold particles per µm^2^ were counted for areas, either placed arbitrarily on the sectioned cells or areas placed on birefringent bodies. The ADY17806 antiserum resulted in a labeling with values of 12 and 231 gold particles per µm^2^ in mean comparing arbitrarily cell areas and birefringent bodies. The birefringent bodies thus showed a 20–30-fold higher labeling in comparison to other cell areas. The densely packed lamellar structure of the birefringent bodies was not visible in the immunostained sections due to the missing Osmium contrast. However, membrane-loop structures were detectable at the endings of the bodies (arrowheads Fig. 11b, d). When the pre-immune sera were used instead of the antisera, a weak background labeling of 0.29 and 0.08 gold particles per µm^2^ in mean was registered on arbitrarily cell areas for AEA49880 and ADY17806. The birefringent bodies showed a similar weak labeling background when using the pre-immune sera (shown for the AEA49880 pre-immune serum in Fig. 10d, not shown for the ADY17806 pre-immune serum).

## Discussion

### The ultrastructure of the birefringent bodies

Since their first description by Dodge and Crawford (1971), exclusively Ammermann et al. (2017) have further characterized the birefringent bodies of *Oxyrrhis marina*. The authors investigated the predatory behavior of *Oxyrrhis marina* feeding on the prasinophyte *Pyramimonas grossii* and registered the fraying form of these bodies. The bodies were located within the food vacuole, often in close proximity to the cytoplasm or clinging to lipid droplets. Elongated-shaped bodies were mainly found, but sickle- and ring-shaped ones were also observed. The elongated bodies measured up to 2 μm in length and 240 nm in width. The width of the sickle-shaped ones was somewhat larger, whereas the overall length of the circle-shaped ones measured up to 6 μm. The edges of the bodies often resembled loop-like, vesicular structures when they peeled off. They measured approx. 16–22 nm in width. Cross-sectioned bodies consisted of highly ordered and densely packed lamellae. They showed a regular striation of rather strong and weak electron-dense lines separated by electron-translucent lines along their longitudinal axis. FFT analysis revealed lattice distances of 11 and 5.5 nm for the densely packed lamellae (Ammermann et al. 2017). In the current study, a lattice distance of 6.1 nm was measured which is in the range of the 5.5 nm value measured earlier. The rather faint and oblique running striation, which was registered beside the longitudinal striation at higher magnification by Ammermann et al. (2017), was observed, too. It formed an angle of approx. 60° with respect to the longitudinal striation. FFT analysis and profile plots revealed a periodicity of 7.5 nm for this lattice pattern. Lattice distances of 7.2–7.8 nm were measured for the highly ordered, honeycombed structures of a birefringent body, which was sectioned rather oblique or almost close to the surface of the outermost lamella (see Fig. 4b). The results obtained from freeze-fractured birefringent bodies in the current study are in line with these results. Freeze-fractured bodies were composed of highly ordered and densely packed lamellae, which were composed of highly ordered, crystalline arrays of particles. The measured lattice distances of 7.0–7.6 nm were similar to those measured for the honeycombed arrays. The bodies seemed to be dimensionally stable, as those isolated by flotation gradient centrifugation did not significantly differ from those within cells with respect to densely packed lamellae and crystalline arrays of particles.

### Are the birefringent bodies waste, and how do they develop?

Birefringent bodies were never observed among the excretion products outside of *Oxyrrhis marina* cells, neither by Ammermann et al. (2017) nor in the current study. This finding led to the conclusion that the bodies most likely do not represent accumulated waste products. Based on the results from ultrathin-sectioned cells, it can be assumed that they originally arise from cytoplasmic membranes enclosing the food vacuoles by evagination into the lumen of the vacuoles, accompanied by stacking in a meandering pattern and densely packaging into highly ordered and densely packed lamellae.

### What tells us the absorbance spectrum?

Normally, laser scanning microscopy is not the method of choice for measuring absorbance spectra but turned out to be of great help in the current study as it provided first evidence on the feasible constituents of the birefringent bodies. Based on the absorbance spectrum registered for the slightly reddish areas, we assumed that these areas are composed of rhodopsins and that the method worked properly could be demonstrated using the PE–565 extract of a cryptophyte. The absorbance maximum was rather broad, showing a faint peak at 540 nm. Rhiel et al. (2020a) registered a faint maximum at 520 nm for rhodopsin-enriched fractions of *Oxyrrhis marina*. Janke et al. (2013) cloned, expressed, and purified the wild-type protein and point-mutated derivatives of the most abundant rhodopsin (ABV22426) of the *Oxyrrhis* strain investigated in yeast and registered an absorbance maximum at 519 nm for the recombinant wild-type protein. The authors state that the absorbance maximum was susceptible to pH, ionic strength, and anions added. Kikuchi et al. (2021) characterized an additional *Oxyrrhis* rhodopsin, AIN36546, by an *E. coli* cell expression system and registered an absorbance maximum at 533 nm. The discrepancy of approximately 10–20 nm measured in the current study might be due to several reasons, i.e., by the different experimental setups used. The cells were fixed in f/2 medium and afterwards washed and stored in TBS for absorbance spectroscopy in the current study. Thus, the spectrum was recorded in situ from areas within fixed cells. Further it has to be considered that not only transmitted light from the birefringent bodies was detected with the applied microscopical method but also “contaminating” light from cell areas below and above the bodies. All steps during the isolation of rhodopsin-enriched fractions were done with distilled water in the study of Rhiel et al. (2020a), and Janke et al. (2013) and Kikuchi et al. (2021) used rhodopsins derived from expression systems. The applied methods using laser scanning microscopy for absorbance measurement nicely worked in the current study and resulted in an absorbance spectrum, almost typical for rhodopsins.

### The reddish areas, the birefringent bodies, and rhodopsins

Based on size and morphology, it could be assumed that the slightly reddish areas represent birefringent bodies and that they are composed of rhodopsins. The registered absorbance spectra of the reddish areas and the ultrastructural findings obtained for the birefringent bodies also pinpoint towards the same direction. The lattice patterns registered for the honeycombed structure by ultrathin sectioning and for the lamellae of the birefringent bodies by freeze-fracturing evidence highly ordered crystalline arrays of the compounds constituting them. The measured periodicities of 7.0–7.8 nm are similar to values registered for (proteo)rhodopsins. Shastri et al. (2007) expressed a proteorhodopsin of an uncultured marine gamma proteobacterium EBAC31A08 in *E. coli*, reconstituted the recombinant proteorhodopsin into proteoliposomes, and registered the formation of two-dimensional crystals with hexagonal protein packing by electron microscopy. The authors assumed a ring-shaped oligomeric assembly of the proteorhodopsin and calculated that the protomers assembled into ring-like structures with average diameters of 4.2 nm. Using atomic force microscopy, Klyszejko et al. (2008) showed that these complexes were formed by hexamers and pentamers. Blaurock and Stoeckenius (1971) determined the X-ray diffraction pattern of purple membrane fragments being solely composed of bacteriorhodopsin. The authors registered planar hexagonal arrays and calculated a distance of 6.3 nm between the centers of adjacent hexagons. Thus, based on the results obtained by absorbance spectroscopy and ultrastructure, it could be assumed that the birefringent bodies are composed of rhodopsins.

Investigations on the subcellular location of rhodopsins of *Oxyrrhis marina* are spare and gave rise to somewhat contradictory results. Slamovits et al. (2011) raised an antiserum against the same identical 14 C–terminal amino acid residues of ADY17806 used in the current study. Applying immunofluorescence light microscopy, the authors registered that the labeling occurred unevenly in the cytosol. In many cells, a single ring-like structure was observed. Hartz et al. (2011) demonstrated the presence of the two rhodopsins ABV22426 and ABV22427 exclusively in the outer plasma membranes of *Oxyrrhis* cells. Currently, no other rhodopsin of *Oxyrrhis marina* has been investigated with respect to its subcellular localization. Rhiel et al. (2020b) raised an antiserum against a rhodopsin-enriched fraction consisting of several rhodopsins. The authors registered strong labeling of the cell periphery by immunofluorescence light microscopy. Additionally, some cell internal structures became labeled. Immunoelectron microscopy of freeze-fractured cells revealed that most likely the membranes of the amphiesmal vesicles were labeled at the cell periphery, while the cell internal label seemed to originate from the food vacuoles. The labeling of birefringent bodies was not registered in this study, most likely due to the fact that birefringent bodies are rarely found within freeze-fractured cells and that a thoroughly inspection of the replica is needed to document at least some of them.

The immunolabeling experiments of the current study using the antisera directed against the rhodopsins AEA49880 and ADY17806 of *Oxyrrhis marina* finally confirmed that both are localized within the birefringent bodies. This result is in line with the findings of Slamovits et al. (2011) who found no evidence that the rhodopsin was localized within the plasma membrane, the nucleus, or mitochondria, and therefore supposed the endomembrane system to harbor rhodopsins. The ring-like structures registered by Slamovits et al. (2011) might be the sickle- and ring-shaped birefringent bodies registered by Ammermann et al. (2017). They might also represent food vacuoles, which became labeled in the study of Ammermann et al. (2017), but this was not registered in the current study. In summary it can be stated that the rhodopsins AEA49880 and ADY17806 are major components of the birefringent bodies.

### What are the functions of the birefringent bodies?

Both rhodopsins deviate significantly with respect to amino acid sequence from one another. Guo et al. (2014) showed phylogenetic affiliations of these two rhodopsin sequences with the proton pump and sensory types, respectively. Thus, ADY17806 rather represents a H^+^ pumping-type rhodopsin, while AEA49880 most likely belongs to the sensory-type rhodopsins (see also Figs. 1 and 2a of Slamovits et al. (2011), and Table 2 of Rhiel et al. (2020a)). Therefore, with respect to function, the birefringent bodies of *Oxyrrhis marina* can be interpreted in different ways. They might function as active, “acidifying organelles” which are necessary for the acidification of food vacuoles, as “H^+^ pumping and thus ATP-generating organelles”, or as “light-sensory organelles,” necessary to trace pigmented prey organisms. The latter assumption supports the finding of Hartz et al. (2011), who showed in photophysiological experiments that, based on rhodopsins, *Oxyrrhis marina* was able to orient towards light. The authors further mentioned that *Oxyrrhis marina* may be able to use them to detect algal prey, based on the prey’s chlorophyll autofluorescence. The birefringent bodies might also perform two or even all three functions simultaneously or function as a “silent reserve” of non-active rhodopsins, which are not needed at that time in this interesting dinoflagellate. Further studies are needed to elucidate the real function of the birefringent bodies of *Oxyrrhis marina*.
